# Influence of surfactant on the permeability at different positions of a leaching column

**DOI:** 10.1371/journal.pone.0274073

**Published:** 2022-09-21

**Authors:** Ai Chun-ming, Sun Ping-ping, Yin Sheng-hua, Chen Xun, Zhong Jia-lin

**Affiliations:** 1 College of Safety Science and Engineering, Liaoning Technical University, Huludao, China; 2 Key Laboratory of Thermal Disaster and Prevention, Ministry of Education, Huludao, China; 3 University of Science and Technology Beijing, Beijing, China; 4 Geological Party No.243, CNNC, Chifeng, Inner Mongolia, China; Tribhuvan University, NEPAL

## Abstract

To solve the problems of poor permeability and low leaching rate in ore heap leaching, solid surface physical chemistry, seepage mechanics theory for porous media, CT scanning and SEM were used to carry out column leaching tests with a homemade segmented removable plexiglass column; the variation law for the permeability coefficients of each segment of the leaching column before and after leaching was analyzed. The experimental results showed that there was little difference in the permeability coefficient of ore at different heights before leaching. After leaching, the permeability coefficients were unevenly distributed along the column height, and the lowest value was located at the bottom of the leaching column. The addition of surfactant provided an obvious improvement in the permeability of the leaching column. The permeability coefficient at the bottom of the leaching column was 6% higher than that of the control group. At the same time, the addition of surfactant increased the leaching rate of ore by nearly 10%. A theoretical analysis showed that the surfactant improved the permeability of ore heaps mainly by preventing physical blockage by fine particles and inhibiting deposition of chemical products.

## 1. Introduction

Heap leaching of ore has been widely used because of the simple equipment, low cost and low energy consumption involved [1–3]. In the heap leaching process, solution transport of target minerals and useful components out of the ore heap must be completed by seepage. At the same time, the permeability of the ore heap directly affects the uniformity of solution distribution in the ore heap, and leaching dead corners and blind areas impede recovery of the target metal [4–7]. The worse the permeability is, the lower the recovery rate. The poor permeability of ore heaps has become a major problem restricting the development of heap leaching technology.

The permeability of ore heaps is an important factor affecting ore heap leaching. Scholars [8, 9] have carried out relevant research on the relationships between ore heap structure, the leaching process and ore heap permeability. Zhang [10] investigated the effect of three types of grain size gradation on porosity and pore size distribution using the bulk density and the computed tomography (CT) scanning methods. Robertson [11] found through numerical simulations that the heap height varies with the progress of heap leaching. The dissolution of minerals leads to ore disintegration, which results in a decrease in ore heap height and then a decrease in the porosity and permeability of ore heaps. Ilankoon and Neethling [12] carried out solution seepage tests with uniform particles and graded particles and analyzed the effects of particle gradation on solution seepage from a heap leaching system. Lu et al. [13] used acid curing and agglomerating technology to improve the average heap permeability effectively. Ghasemzadeh et al. [14] studied the effect of sulfuric acid on hydraulic mechanical properties through friction angle and slake durability indices. Dhawan et al. [15] proposed that the curing and associated precipitation of compounds such as sulfide and calcium sulfate can be important in ore heap leaching.Therefore, a series of measures should be taken to reduce the precipitation in the process of heap leaching.

To improve the permeability of heap leaching systems, scholars have proposed a variety of measures designed to improve the permeability of ore heaps with physical and chemical approaches, mainly including fine ore granulation technology [16], ore grading of heap buildings [17], mechanical loosening of heaps [18], and surfactant addition [19, 20], which have provided good results. Among them, surfactant addition gives the solution a high surface activity and also leads to good diffusion and permeability. The solution can penetrate quickly into the inner surfaces of pores and fissures in ore particles. At present, surfactants have been applied in the process of metal ore leaching. They show remarkable effects and broad development prospects in improving ore heap permeability and ore leaching rate.

## 2. Experimental

### 2.1 Experimental ore

#### 2.1.1 Test ore sample

The test ore sample was taken from a copper mine in Yunnan Province. The heap leaching method was used to recover copper. The ore was an alkaline oxide ore with a high mud content, and the copper grade was 1.26%. In the production process, the problem of poor permeability in ore heaps led to the failure of ore recovery.

The raw ore had high contents of fine ore and mud ore, and the proportion of clay minerals such as sericite and kaolinite exceeded 25%, and the ore contained 40% limonite, which was easily silted. The mud content was large, and the heap leaching permeability was poor. According to Table 1, the mineral composition of alkaline gangue in the ore accounted for 19.65% of the total, and the ore showed high acid consumption during leaching, which resulted in chemical precipitation and pore blockage in the leaching process.

**Table 1:** Chemical contents of ore.

chemical composition	Cu	Fe_2_O_3_	MgO	CaO	SiO_2_	Al_2_O_3_	Zn	S	As	WO_3_
content/%	1.26	28.96	1.39	11.48	47.97	7.82	0.21	0.56	0.16	0.19

#### 2.1.2 Ore particle sizes

**Fig 1:**
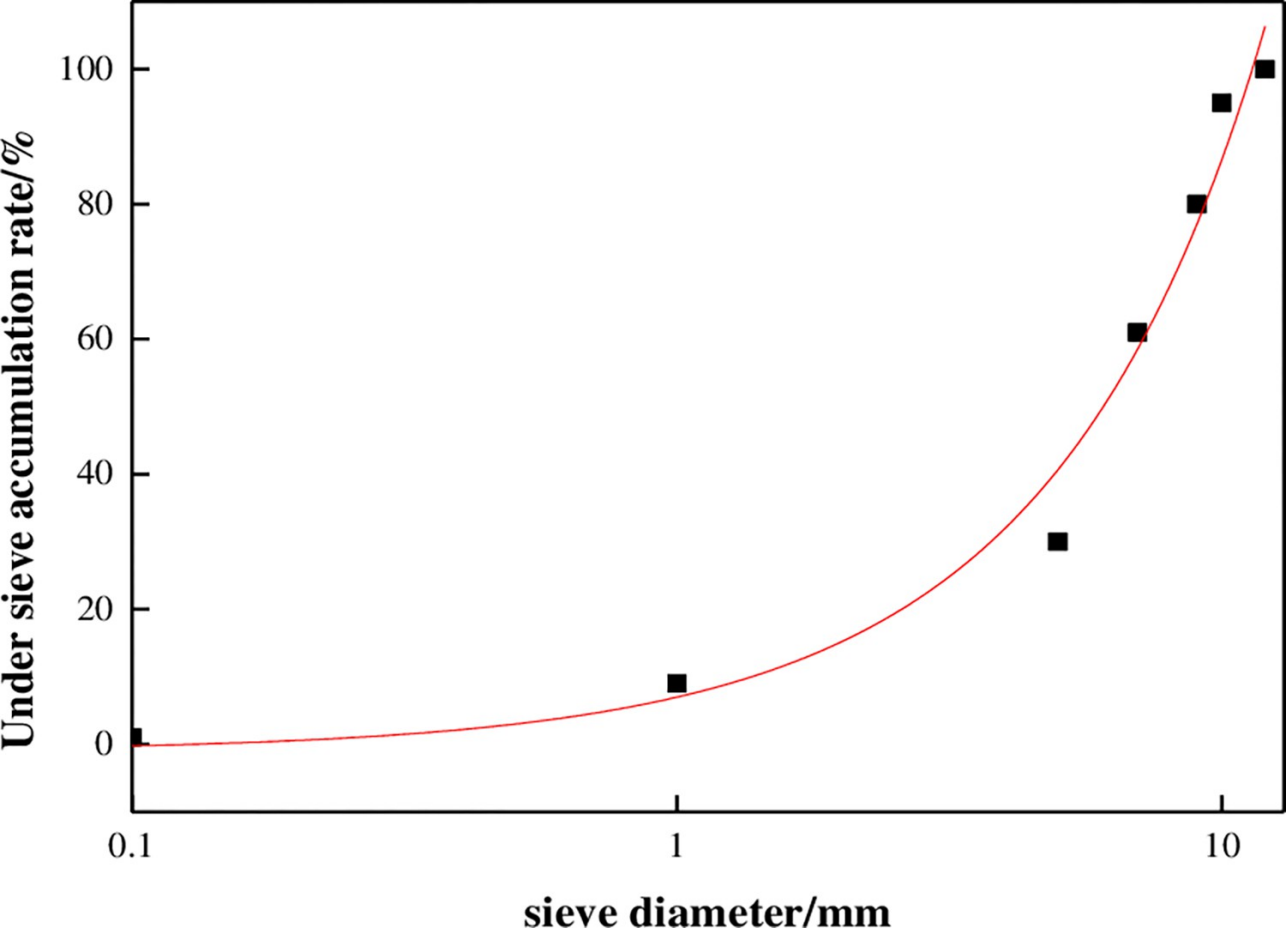
Sample grading curve.

The ore was put into a standard analysis sieve and placed on a shaker for 15 min to obtain the particle size analysis curve, as shown in Fig 1.

The nonuniformity coefficient is:

$$C_{u}=\frac{d_{60}}{d_{10}}=4.85$$ (1)

The curvature coefficient is:

$$C_{c}=\frac{d_{30}^{2}}{d_{60}d_{10}}=2.20$$ (2)

The average particle size is:

$$d_{cp}=\sum_{n=0}^{n}\frac{d_{i}+d_{i+1}}{2}\cdot a_{i}=4.95$$ (3)

where *d_60_*, *d_30_* and *d_10_* are the corresponding particle sizes for 60%, 30% and 10% of the material accumulated during sieving; *d_i_* and *d_i+1_* are the first and last particle sizes of group *i*; and *α_i_* is the mass distribution frequency of the group *i* particle size range.

The copper ore grading curve is concave and conforms to a B-type particle structure [21]. In this type of granular material, the skeleton position of coarse particles is relatively fixed, and the migration of fine particles easily leads to pore blockage.

### 2.2 Testing device

**Fig 2:**
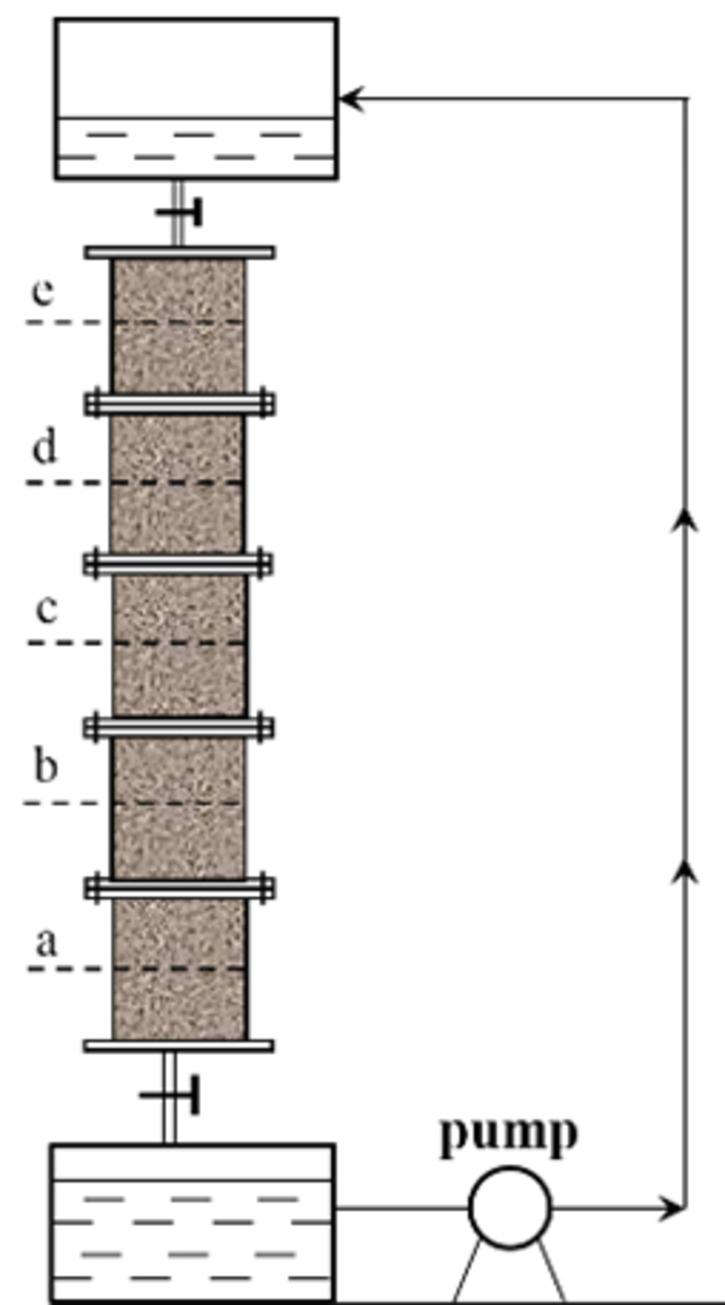
Schematic diagram of the test device.

**Fig 3:**
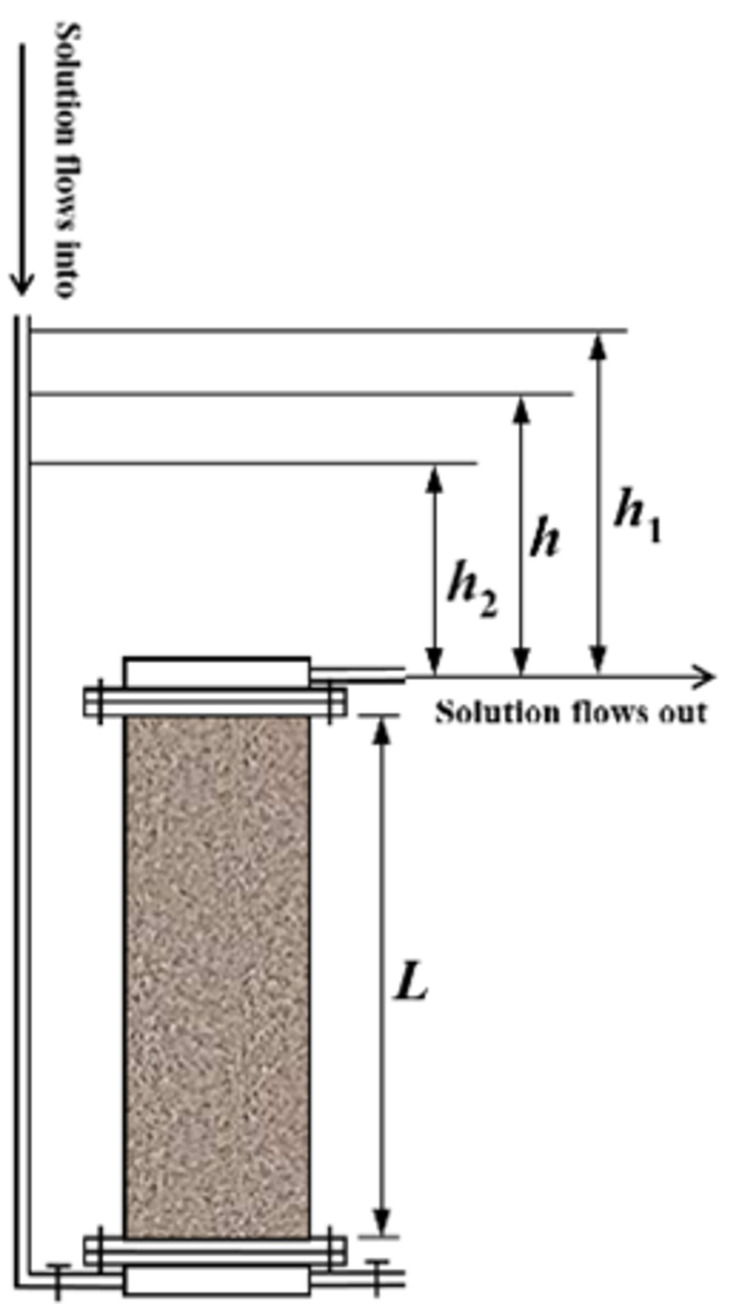
Details of the test device.

**Fig 4:**
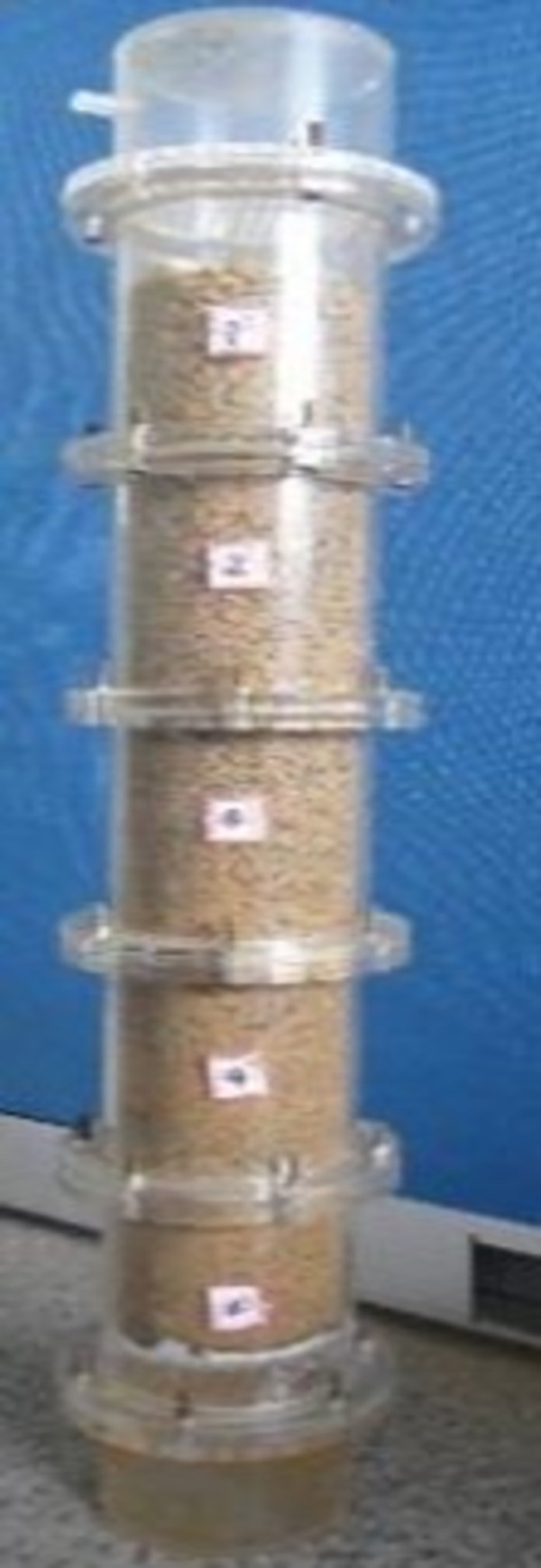
Physical diagram of the test device.

To study the influence of surfactant on permeability during ore leaching, a self-developed device was used to measure the permeability coefficients of ore pillars. The test device is shown in Figs 2–4.

The test device was composed of an ore column, an upper liquid level tank, a lower liquid level tank and a circulating pump. The ore column was made of five removable plexiglass columns with inner diameters of 60 mm and heights of 100 mm, which were connected by flanges and bolts. The upper and lower parts of the device were provided with outlet and inlet devices, which were flange connected, as shown in Fig 4.

### 2.3 Permeability measurements

The height of the device used for measuring the permeability coefficient can be arbitrarily changed as necessary. The permeability coefficients at different heights can be measured in the process of ore leaching. The device can meet the requirements of different heap heights.

At any time *t* during the penetration test, the water level of the variable head was *h* (the height difference between the water surface of the inlet pipe and the water outlet). According to Darcy’s law, the velocity of water in laminar flow at the outlet is:

v=k⋅i (4)

where *k* is the permeability coefficient and *i* is the hydraulic gradient.

The hydraulic gradient can be expressed as:

$$i=\frac{h}{nL}$$ (5)

where *h* is the height of the water head; *L* is the height of a pillar to be measured; and *n* is the number of pillars to be measured.

If the sectional area of the pillar is *A* and the flow rate within time *t* is *Q*, then:

$$\frac{dQ}{dt}=vA=k\frac{h}{L}A$$ (6)

Suppose that the cross-sectional area of the inlet pipe is *a* and the water level in the pipe drops by *dh* within time *dt*; then, the flow is:

dQ=−adh (7)

Combining Eq (3) and Eq (4) and integrating gives:

$$-\int_{h_{1}}^{h_{2}}\frac{dh}{h}=\int_{t_{1}}^{t_{2}}k\frac{A}{La}dt$$ (8)

The permeability coefficient *k* of the pillar can be obtained:

$$k=\frac{aL}{A(t_{2}-t_{1})}\ln\frac{h_{1}}{h_{2}}$$ (9)

where *t_1_* and *t_2_* are different timing moments and *h_1_* and *h_2_* are the water heads for pillar matching at *t_1_* and *t_2_* respectively.

### 2.4 Solution preparation

In this test, sulfuric acid solution was used as the leaching agent, sodium dodecyl sulfate (anionic) was selected as the surfactant, and the relative molecular weight was 288.

Two kinds of leaching solutions were prepared: 1) 20 g/L sulfuric acid solution and 2) 20 g/L sulfuric acid + 0.008 mol/L sodium dodecyl sulfate solution. The amount of surfactant added was determined by a shaking flask test in the early stage.

### 2.5 Test conditions

Two groups of column leaching tests were carried out, The concentration of leaching solution and the type of surfactant was determined according to the previous corresponding experiments [22, 23], the specific grouping was shown in Table 2. The weight of the assembled ore was 1.528 kg (including the filter layer). According to the actual production parameters of the mine, the spraying intensity was 40 L/(m^2^ h), and the cycle frequency was once every 24 h. The column immersion test lasted for 28 days.

**Table 2:** Experimental grouping.

Number	Sulfuric acid concentration (g/L)	Surfactant concentration (mol/L)
A	20	0
B	20	0.008

### 3. Experimental results

#### 3.1 Changes in sample pore structures

The pore structures before and after the test were observed by X-ray CT scanning technology. The CT scanning equipment used in this experiment was a μCT225KVFCB-type high-precision microscopic CT test system, which had a discharge point multiple of 1-400 times. The specimen size range was φ 1-50 mm, and the maximum spatial resolution was 0.485 μm.

**Fig 5:**
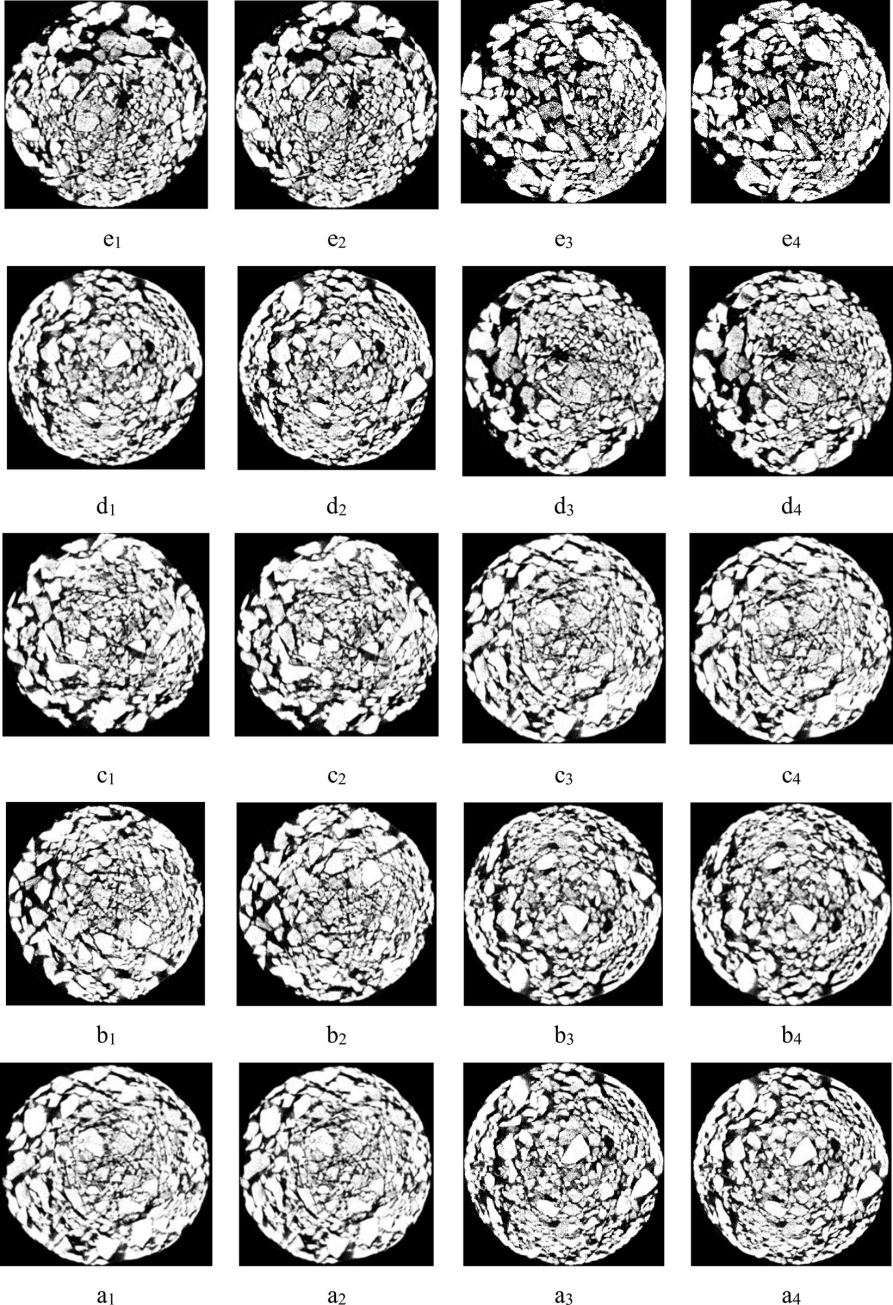
Sample structures of groups A and B before and after testing.

The marked positions on the test columns of groups A and B were scanned by CT. The scanning parameters were set as follows: the tube voltage of the CT testing instrument was 120 kV, the current was 160 μA, the magnification factor was 4.14 times, and the resolution of the images in the XY direction was 46.86 μm. The 2D cross-sectional images of the before and after pillars from groups A and B were reconstructed, as shown in Fig 5. Because the density difference between ore particles and pore area were obvious in the column leaching system, the CT scanning image was segmented by ImageJ software.

Fig 5 shows CT scanning diagrams of sample structures before and after group A and B tests. Letters a, b, c, d and e represent the middle positions of the first section to the fifth pillars, respectively, as shown in Fig 2. Numbers 1 and 2 represent data taken before and after the group A test, respectively, and 3 and 4 represent data taken before and after the group B test, respectively.

**Fig 6:**
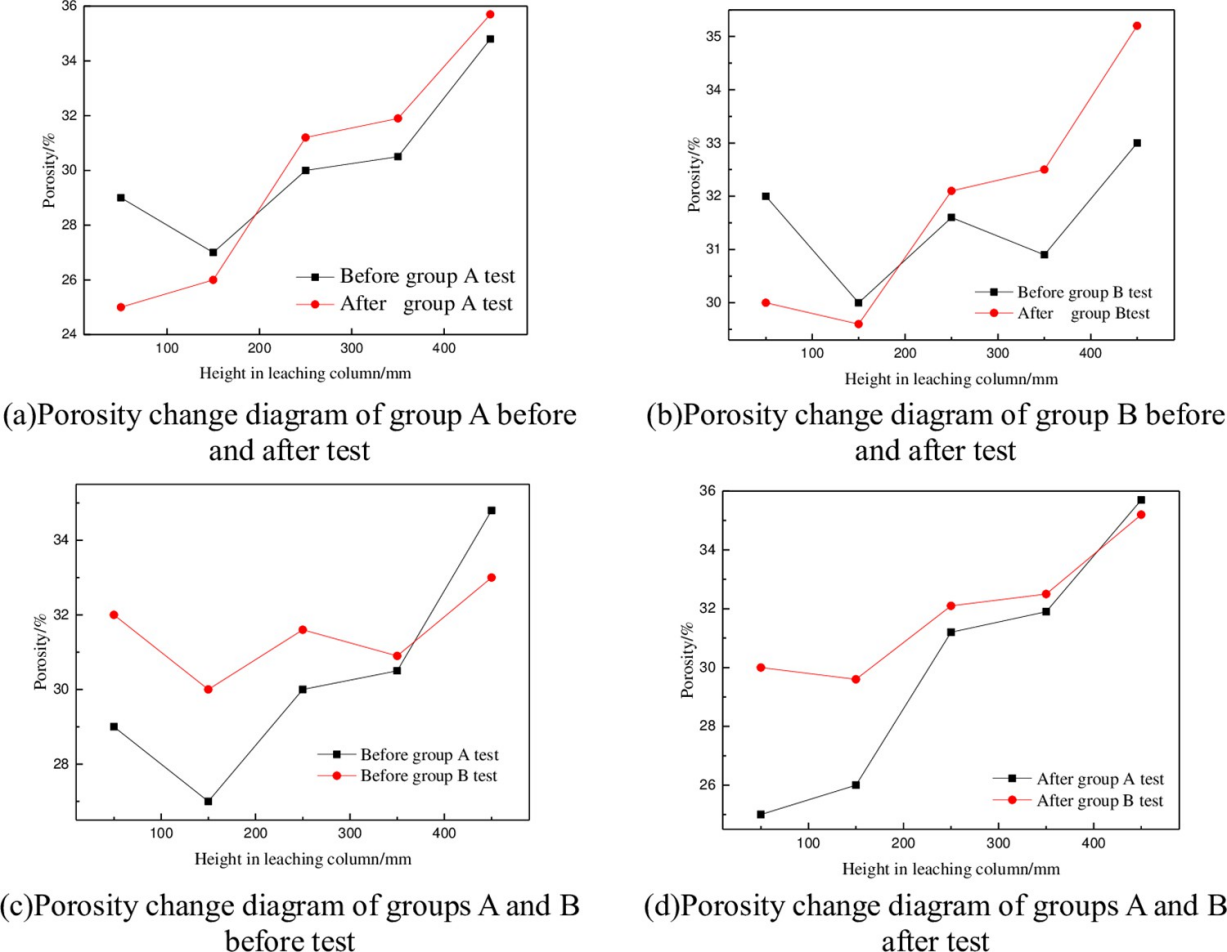
Changes in ore porosity before and after group A and B tests. (a)Porosity change diagram of group A before and after test. (b)Porosity change diagram of group B before and after test. (c)Porosity change diagram of groups A and B before test. (d)Porosity change diagram of groups A and B after test.

After the CT scan images were processed, the porosities of each position before and after test groups A and B were calculated according to Eq (10), and porosity change curves were drawn for different heights, as shown in Fig 6.

$$\phi =1-\frac{\rho_{c}}{\rho_{s}}$$ (10)

where *ρ_c_* is the sample density and *ρ_s_* is the particle density of the sample.

Fig 6(A) and 6(B) shown that after leaching, the porosity at positions c, d and e of group A and B ore columns increased compared with that determined before the test, while the porosities at positions a and b decreased, and the decrease was largest at a height of 50 mm. According to Fig 6(C) and 6(D), compared with that for group A, the porosity fluctuation range of group B was smaller, especially near the bottom area; the porosity change was not obvious.

**Fig 7:**
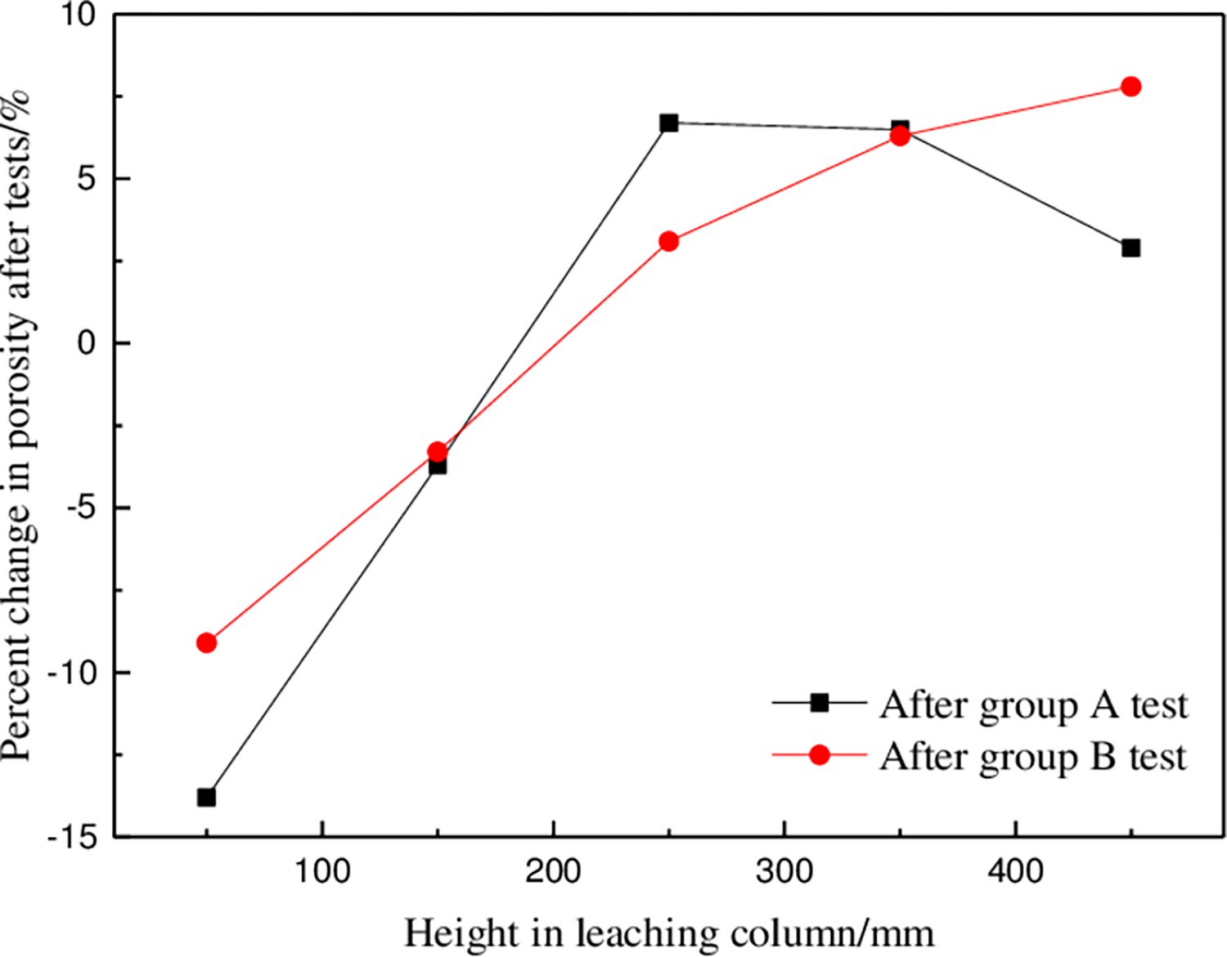
Schematic diagram of percentage change of ore porosity after group A and B tests.

It can be seen from Fig 7 that the change percentage of ore porosity in group B was greater at a and e than that in group A after the test, indicating that the action positions of surfactant added in group B are mainly at the top and bottom of the leaching column. Surfactant reduced physical and chemical deposition phenomena in the heap leaching process; the particles were in a dispersed state and showed no aggregation or connections. The increase in ore porosity was conducive to seepage of leaching solutions between ores and improved the permeability of heap leaching.

### 3.2 Change in permeability coefficient

**Fig 8:**
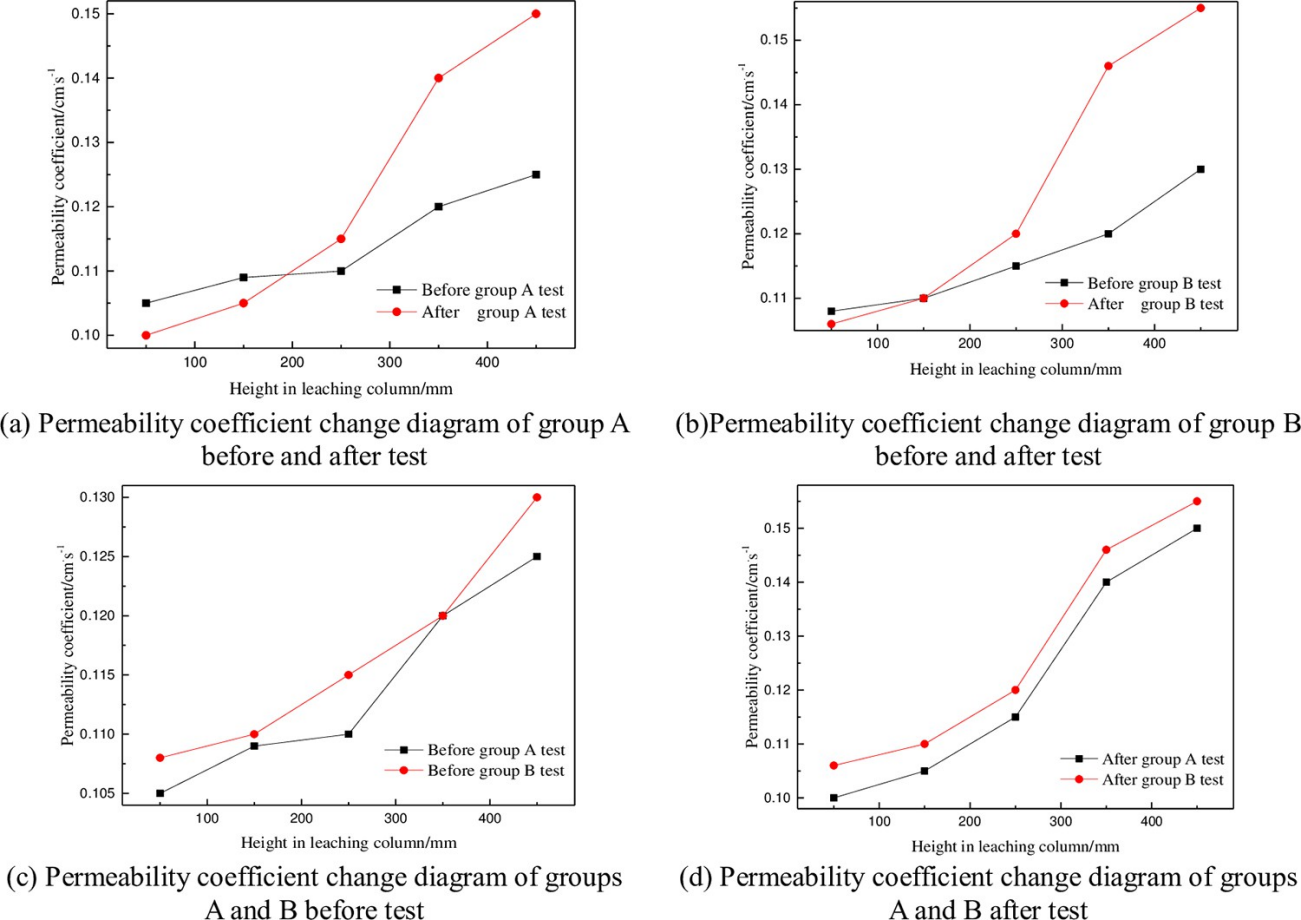
Change diagrams for permeability coefficients in the column immersion test. (a) Permeability coefficient change diagram of group A before and after test. (b)Permeability coefficient change diagram of group B before and after test. (c) Permeability coefficient change diagram of groups A and B before test. (d) Permeability coefficient change diagram of groups A and B after test.

The permeability of the leaching pillar was measured by the variable head method [24], and permeability coefficient data were measured at different positions of the pillar before and after the test. Eq (9) was used to calculate the corresponding permeability coefficients, as shown in Fig 8.

Fig 8(A) and 8(B) shown that the permeability coefficient of the ore column increased over different ranges in the direction of ore column height for both group A and group B, and the permeability coefficient at position e after the group B test reached 1.5 times that of group A. The permeability coefficients of group A and group B were also significantly different before and after the tests. The permeability coefficients of ore columns a and b in group A were smaller than those before the test, and the permeability coefficients of group B were always greater than before the tests except for position a.

#### 3.2.1 Influence of column height on permeability coefficient

**Fig 9:**
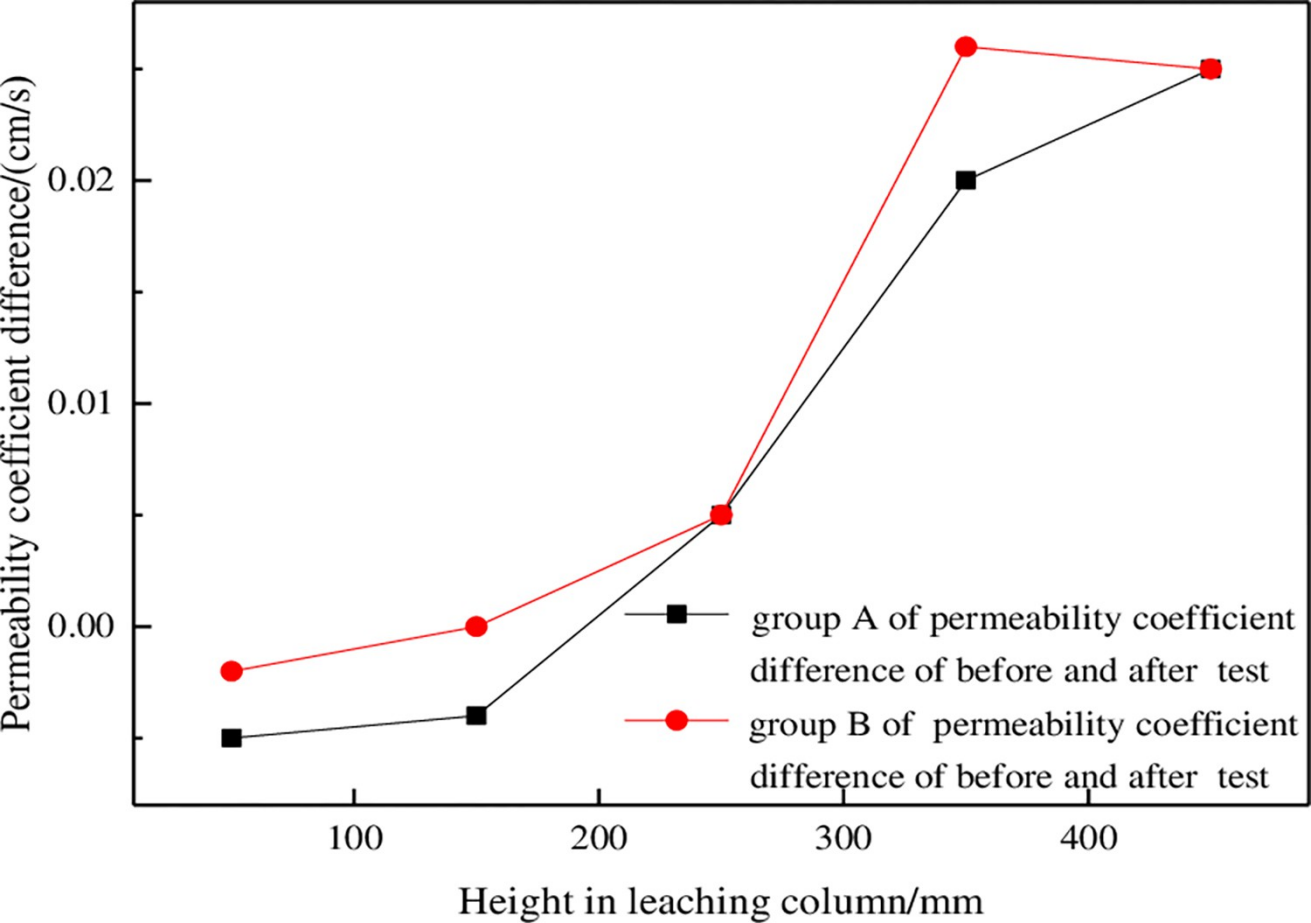
Permeability coefficient differences before and after testing of groups A and B.

The difference in permeability coefficients for groups A and B before and after testing was calculated (after the test - before the test), and difference curves were drawn for permeability coefficients of the ore columns at different heights, as shown in Fig 9. The variation trends for differences in permeability coefficients between the two groups were similar. Whether in group A or B, the differences at a and b were not greater than zero, while the differences at c, d and e were positive, and the differences in permeability coefficients at ore columns d and e were significantly greater than those at a. This shows that in the process of ore leaching, the permeability coefficient of the ore column decreased from the top to the bottom, and the permeability at the bottom of the ore column was the worst, which is mainly due to two reasons:

Physical deposition [25]. The leaching solution flows from top to bottom in the ore column, and this drives the migration of fine particles in the ore. With increasing progress of the leaching reaction, fine particles moved downward and caused local siltation; this blocked leaching solution seepage channels and led to gradual accumulation at the bottom of the ore column.

Chemical deposition [26]. In the process of column leaching, sulfuric acid reacts with acid-consuming minerals such as aluminum, calcium and magnesium oxides in the ore, forming insoluble precipitates such as CaSO_4_ and MgSO_4_; these flow downward with the leaching solution and finally accumulate at the bottom of the ore column, resulting in narrowing of seepage channels, increasing seepage resistance and significant decreases in permeability coefficients.

#### 3.2.2 Influence of surfactant on permeability coefficients

**Fig 10:**
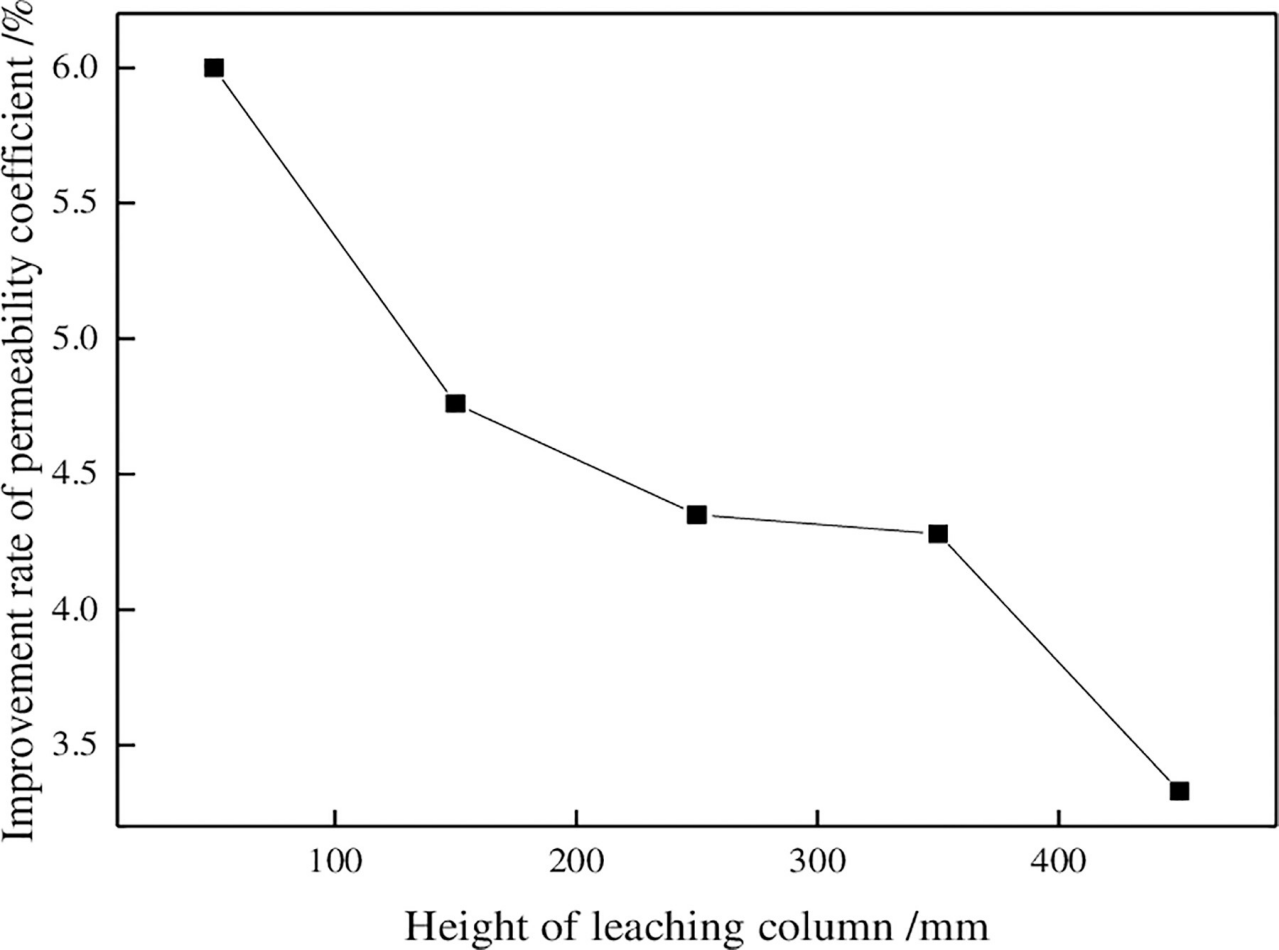
Changes in permeability coefficients with column height.

To analyze the influence of surfactant on the permeability coefficient of the ore leaching test, change curves for increases in permeability coefficients of the ore column with height were drawn, as shown in Fig 10. The improvement of the permeability coefficient was defined as the ratio of the difference between the permeability coefficients after the tests of group A and group B and the permeability coefficient after the test of group A, that is:

$$w=\frac{k_{2}-k_{1}}{k_{1}}\times 100\%$$ (11)

where *w* is the degree of increase in the permeability coefficient, in %; *k_2_* is the permeability coefficient after testing of group B, in cm/s; and *k_1_* is the permeability coefficient after testing of group A, in cm/s.

Fig 10 shown that the improvement in the permeability coefficient of the ore column decreased with increasing height. In ore columne, the improvement rate of the permeability coefficient of group B was only 3.33%. At a, the improvement rate of the permeability coefficient reached the maximum at almost twice that of e. It can be seen from Fig 7(C) and 7(D) that the permeability coefficient of groups A and B has A similar change trend after the test, but group B was always larger than group A. Because adding surfactant effectively improved the permeability of ore column. The permeability of the ore column can be improved mainly in the following ways:

1. The migration of fine particles leads to reduction in seepage channels, and the surfactant disperses the ore particles, which places the particles in a relatively stable suspension or dispersion state and leaves them unable to agglomerate; this expands the seepage channels and stabilizes the seepage burst of the leaching solution.
2. With increasing progress of the leaching reaction, surfactant adsorption on the surface of the ore, which causes the surfaces of the ore particles to exhibit the same electrical repulsion, hinders the aggregation of fine particles and is conducive to the flow of leaching solution in the ore pores. At the same time, it is not conducive to scaling caused by chemical reaction products and ensures a smooth seepage channel.

### 3.3 Change in ore leaching rate

**Fig 11:**
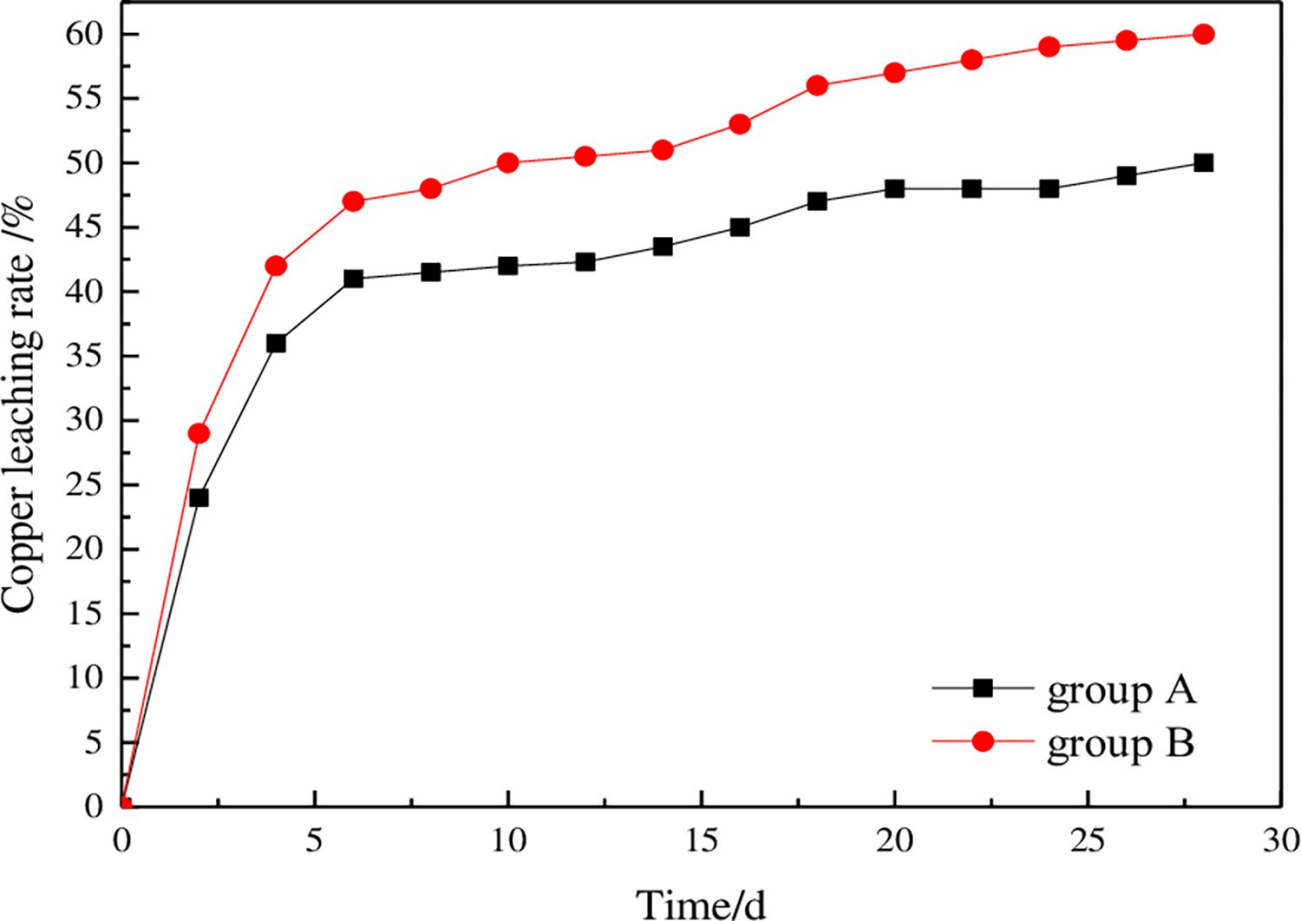
Change curves for copper leaching rates in the column leaching tests.

The ore leaching test was carried out for 28 days. The copper ion content in the leaching solution was detected every 2 days, The copper leaching rate was calculated according to Eq (12). The change curve for the copper leaching rate with time is shown in Fig 11.

$$Cu_{l}=\frac{a_{i}V_{i}}{QC}\times 10^{4}$$ (12)

where *Cu_l_* - the copper leaching rate, %; *a_i_* - qualified liquid concentration of grade *i* reaction, g/L; *V_i_* - qualified liquid volume of grade *i* reaction, L; *Q* - ore mass before leaching, g; *C* - the copper content in ore before leaching, %.

In the initial stages of the column leaching test, most of the ore was leached, and the leaching rate increased rapidly. With continuous cycling of the leaching reaction, the leaching rate for copper ions decreased, and changes in the curve tended to be minimal. In later stages of leaching, the leaching rate for copper hardly changed until the end of the experiment. The leaching rate for copper ore was increased from 50% to 60% by adding surfactant into the leaching solution.

According to the figure, addition of surfactant improved the permeability of the ore and also improved the leaching rate. First, the improvement in the permeability of the leaching column indicated that the flow of the leaching solution in the ore was smoother, which was conducive to contact between the leaching solution and the target mineral for chemical reactions. Second, the target minerals were dissolved in the solution, and the useful components were transported over time, which promoted external diffusion in the leaching process and improved the leaching speed. Finally, the ore column had good permeability, not only in the vertical direction but also in the horizontal direction, which made the leaching solution more evenly distributed in the ore and the leaching reaction more efficient.

## 4. Surfactant influenced the mechanism of seepage

### 4.1 Preventing physical blockage of fine particles

#### 4.1.1 Physical blockage caused by fine particle migration

**Fig 12:**
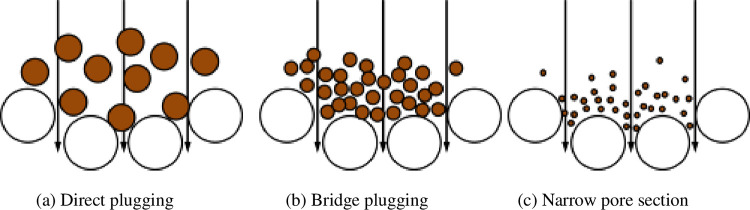
Schematic diagrams of particle migration and deposition. (a) Direct plugging. (b) Bridge plugging. (c) Narrow pore section.

When the leaching solution flowed from top to bottom in the ore column, part of the fine ore and clay migrated to form local or large-scale aggregates, which blocked the liquid flow channels of ore particles and hindered uniform flow of the solution. The sample pore structure was affected by fine particle migration and sedimentation, as shown in Fig 12. After entering the pore structure, the particles continuously migrated in the direction of pressure reduction. When encountering a channel smaller than the particle size, the particles could not pass and caused blockage, as shown in Fig 12(A). When multiple fine particles reached the pore throat at the same time, "bridge plugging" occurred, and the permeability of the pore structure was reduced (Fig 12(B)). When fine particles entered large-diameter pores, some particles were deposited on the pore surface, reducing the effective seepage channel, as shown in Fig 12(C).

#### 4.1.2 Dispersion of ore particles by surfactants

To better explain the dispersive effects of surfactants on particles, electrostatic stability theory (DLVO theory) was introduced. The basic idea of the theory is that when different charged colloidal particles are close to each other, there are van derwaals attractive and repulsive forces caused by the overlap of electric double layers. The changes in potential energy caused by these two forces as a function of particle distance and relative size determine the stability of the system.

According to the two-electron model, when *s* ≤ *r*, the total potential energy for the interaction between ore particles is:

$$V=\frac{1}{2}\varepsilon r\varphi^{2}\ln[1+e^{\left(-Ks\right)}]-\frac{Hr}{12s}$$ (13)

where *ε* is the dielectric constant; *r* is the particle radius; *φ* is the surface potential; *s* is the surface distance between particles; *K* is the Debye-Hegel constant; and *H* is the Hamack constant.

**Fig 13:**
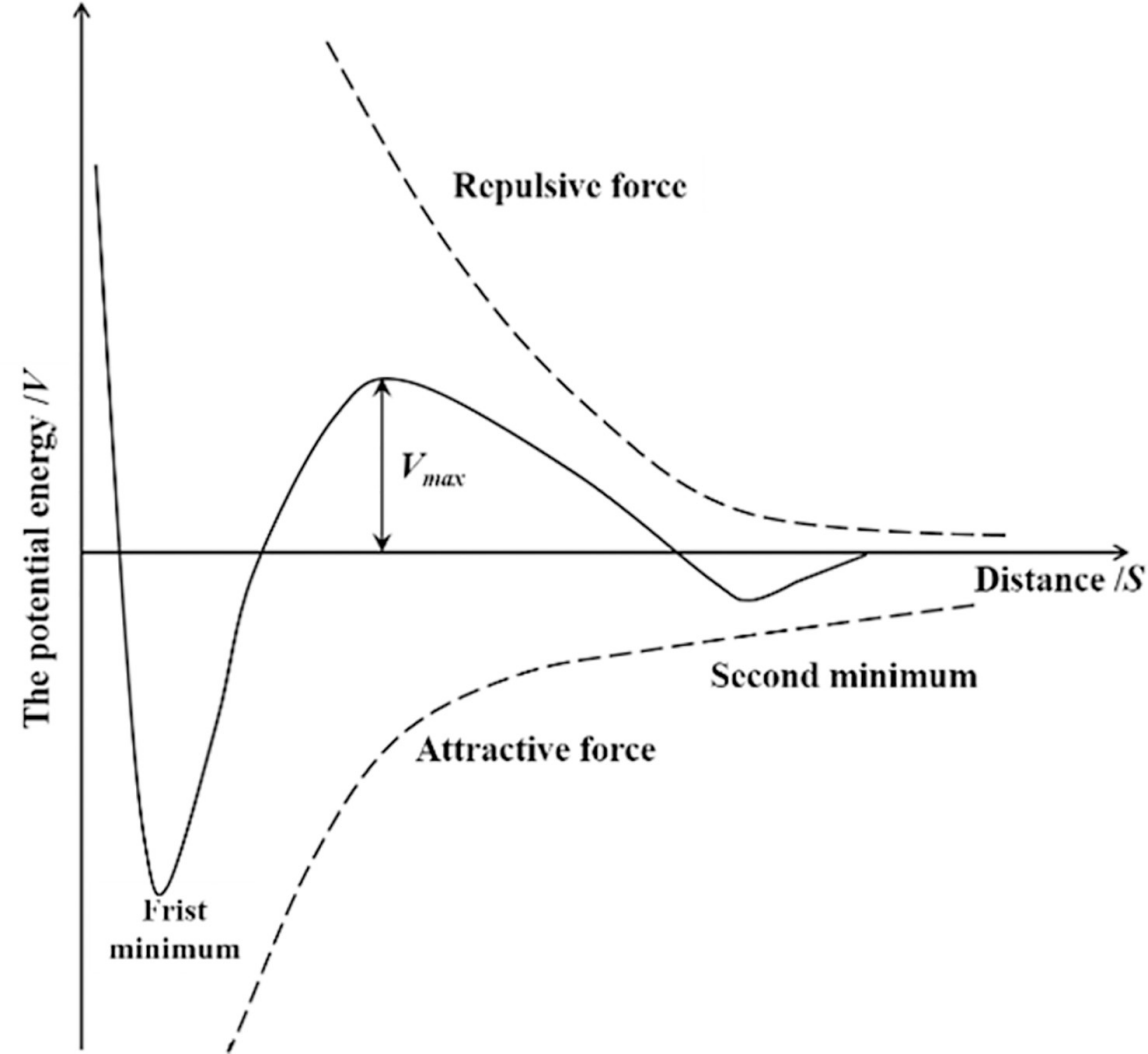
Schematic diagram for total potential energy.

The relationship between total potential energy (*V*) for the interaction between particles and the distance between particles (*S*) is shown in Fig 13. When the distance between particles is large or small, van der Waals attractive forces are dominant, and the particles mainly attract each other. In the intermediate state, the repulsive force is more dominant, and there is a maximum *V_max_* on the potential energy curve; this is the potential energy barrier. The potential energy barrier indicates that the resultant force between ore particles is a repulsive force. If the thermal movements of particles cannot allow them to overcome the potential energy barrier, they cannot aggregate and remain in a stable dispersed state.

According to Eq (13), the total potential energy (*V*) between ore particles is directly proportional to the solid surface potential (*φ*) and electrolyte concentration (*ε*) and inversely proportional to the Hamack constant (*H*). After adding a surfactant, ionization and adsorption increase the double layer potential repulsive forces operating between particles *φ* and *ε*. Additionally, the adsorption layers formed by surfactant on the surfaces of fine particles decrease *H*.

Adding surfactant can improve the total potential energy between fine particles. To achieve agglomeration between particles, a higher potential energy barrier must be overcome, so fine particles are relatively stable in a dispersed state, increasing the porosity at different positions of the ore column (as shown in Fig 6) is conducive to the seepage of leaching solution between ores and improve the permeability of heap leaching.

### 4.2 Inhibition of chemical product deposition

#### 4.2.1 Chemical product deposition process

In the leaching process, sulfuric acid reacts with acid-consuming minerals such as aluminum, calcium and magnesium oxides in the ore and forms insoluble precipitates such as CaSO_4_, which reduces the diameters of seepage channels, increases seepage resistance, and greatly reduces the permeability coefficient. The chemical reaction of Ca^2+^ and SO_4_^2-^ is used as an example to analyze the causes of precipitation.

When the concentrations of Ca^2+^ and SO_4_^2-^ in the solution are higher than the concentrations at dissolution equilibrium, the anions and cations in the solution form ion pairs due to the interactions of charges. The number of ion pairs continues to increase, and they aggregate to form crystal nuclei. After the crystal nuclei continue to grow, they precipitate from the solution and form CaSO_4_ crystals. The crystals diffuse to the structural plane under the influence of a concentration difference and finally cover the structural plane, and the process is controlled by mass transfer. In addition, Ca^2+^ and SO_4_^2-^ ions in the solution also diffuse to the structural plane due to the concentration differences and connect with each other under the influence of charge attraction and form CaSO_4_ crystals attached to the structural plane; this process is controlled by chemical reactivity.

#### 4.2.2 Influence of chemical precipitation on ore leaching permeability

The acidic liquid reacts with the ore, and the ore surface is changed by, e.g., dissolution and disintegration. As the leaching reaction continues, precipitation of calcium and magnesium compounds and iron-containing cement also occurs between particles; this connects loose particles, reduces the overall porosity of the particle group, and leads to poor permeability. The influence of precipitates produced by chemical reactions on ore leaching permeability is mainly caused by three factors:

1. Accumulation and growth of a large number of crystal precipitates eventually connect the ore particles together in the form of cement, which results in the loss of intergranular pores and affects the seepage of solution through the particle group.
2. Crystalline or colloidal substances produced by chemical reactions are deposited on the particle surface, which prevents the solution from contacting useful components.
3. Chemical precipitation gradually occurs in pores or on throat surfaces, and the particles firmly adhere to pore throat surfaces, which hinders continuous infiltration of solution into the ore.

#### 4.2.3 Surfactant prevention of precipitation

In this study, sodium dodecyl sulfate was selected as the anionic surfactant, and it is soluble in water and has a negative charge. Electrostatic repulsion caused the surfactant molecular chain to expand, and charged CaSO_4_ microcrystals were adsorbed by ions with opposite charges. The chain structure of the surfactant adsorbed multiple microcrystals at the same time, reduced the number of microcrystals in solution and reduced the likelihood of precipitation. Adsorption of the surfactant changed the charge distribution on the microcrystalline surface and formed an electric double layer. The microcrystalline surfaces exhibited the same charges, which increased electrostatic repulsions between particles so that the microcrystalline material maintained a stable dispersed state, was suspended in solution and avoided grain aggregation and precipitation.

**Fig 14:**
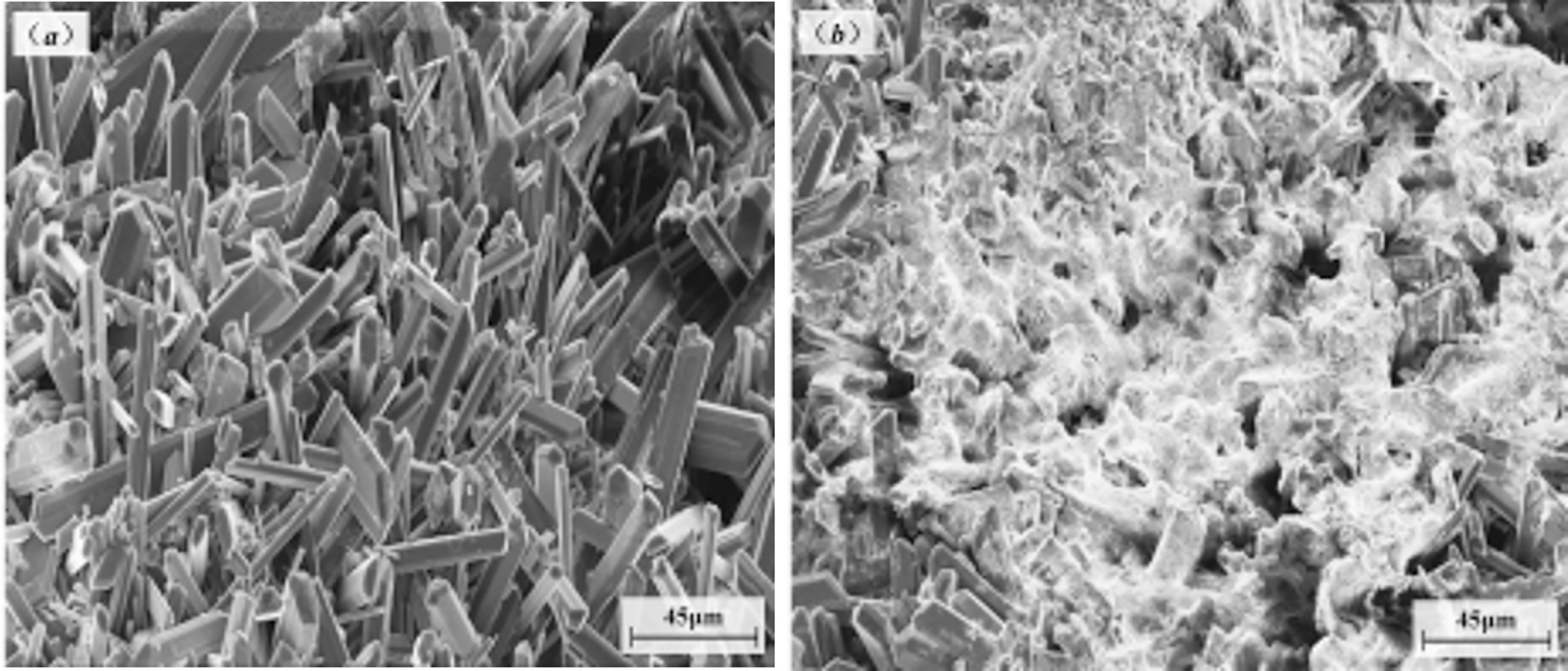
Ore crystal surfaces before and after the addition of surfactant.

The surfactant in the solution was attached to the microcrystals generated, changed the growth mode of the crystals, changed the morphology of the crystals, and inhibited and precluded the growth of precipitate. The SEM results for the ore surface before and after the addition of surfactant are shown in Fig 14.

Without surfactant, the precipitated crystals on the ore surface were well developed and dense typical hexahedral crystals, as shown in Fig 14(A). After surfactant was added, the crystals were irregular, clumpy and relatively loose, and the crystal structure "collapsed", as shown in Fig 14(B). The surfactant disrupted the normal growth rules of precipitated crystals and produced only amorphous particles; this effectively prevented a large amount of crystalline precipitate from adhering to the ore surface and forming a blockage.

The surfactant in the solution adsorbed on microcrystals and increased the repulsive forces between particles so they remained in a stable dispersed state and greatly reduced the possibility of precipitate formation. The surfactant changed the growth mode of crystals, changed the morphology of crystals, made them unable to crystallize, and effectively inhibited the formation of precipitate, increase the permeability coefficient of the ore column, as shown in Fig 9.

## 5. Conclusion

1. The permeability of the ore column is constantly evolving during the leaching process. Before ore leaching, there was little difference among permeability coefficients for ore at different heights, and the permeability coefficient at the bottom was the lowest. After a column leaching test, the permeability coefficients were unevenly distributed along the column height. The permeability at the top was the highest and was increased by 20%, but the permeability coefficient at the bottom was decreased by 4.76%.
2. Addition of a surfactant improved the permeability of ore columns. Surfactants improved the permeability coefficients for various areas of the ore column, especially for the bottom area, and the permeability coefficient was increased by 6%.
3. After adding surfactant, the leaching rate of copper was increased by nearly 10%.
4. The mechanism by which the surfactant improved the permeability of column leaching was clarified. Under the action of hydraulic forces, gravity and chemical reactions, ore particles settled and accumulated at the bottom of the leaching column, resulting in a decrease in the permeability coefficient. Adding surfactant effectively prevented blockage by fine particles, which was caused by physical action, inhibited the deposition of chemical products and improved the permeability and leaching rates of ore columns.

## Supporting information

S1 Data (XLSX)

## References

[1] Ilankoon I M S K, Yuan T, Yousef G, et al. The current state and future directions of percolation leaching in the Chinese mining industry: Challenges and opportunities. Minerals Engineering. 2018, 125:206–222.

[2] Feng S, Li K, Huang Z, et al. Specific mechanism of Acidithiobacillus caldus extracellular polymeric substances in the bioleaching of copper-bearing sulfide ore. PloS one. 2019, 14(4): e0213945. doi: 10.1371/journal.pone.0213945 30978195 PMC6461249

[3] Neira A, Pizarro D, Quezada V, et al. Pretreatment of Copper Sulphide Ores Prior to Heap Leaching: A Review. Metals. 2021, 11(7): 1067.

[4] Petersen J. Heap leaching as a key technology for recovery of values from low-grade ores – A brief overview. Hydrometallurgy. 2016, 165.

[5] McBride D, Ilankoon I, Neethling S J, et al. Preferential flow behaviour in unsaturated packed beds and heaps: Incorporating into a CFD model. Hydrometallurgy. 2017, 171: 402-411.

[6] Van Staden P J, Petersen J. The effects of simulated stacking phenomena on the percolation leaching of crushed ore, Part 1: Segregation. Minerals engineering. 2018, 128: 202–214.

[7] Nkuna R, Ijoma G N, Matambo T S, et al. Accessing Metals from Low-Grade Ores and the Environmental Impact Considerations: A Review of the Perspectives of Conventional versus Bioleaching Strategies. Minerals. 2022, 12(5): 506.

[8] Wang L, Yin S, Wu A. Ore agglomeration behavior and its key controlling factors in heap leaching of low-grade copper minerals. Journal of Cleaner Production. 2021, 279: 123705.

[9] Van Lien T, Dinh T T, Dung N T K. Study on leaching systems and recovery for PALUA–PARONG low grade uranium sandstone ores. Hydrometallurgy. 2020, 191: 105164.

[10] Zhang S, Liu W, Granata G. Effects of grain size gradation on the porosity of packed heap leach beds. Hydrometallurgy. 2018, 179: 238–244.

[11] Robertson S. Development of an integrated heap leach solution flow and mineral leaching model. Hydrometallurgy. 2016, 169:79–88.

[12] Ilankoon I M S K, Neethling S J. Liquid spread mechanisms in packed beds and heaps. The separation of length and time scales due to particle porosity. Minerals Engineering. 2016, 86:130–139.

[13] Lu J, Dreisinger D, West-Sells P. Acid curing and agglomeration for heap leaching. Hydrometallurgy. 2017, 167: 30–35.

[14] Ghasemzadeh H, Pasand M S, Shamsi M M M. Experimental study of sulfuric acid effects on hydro-mechanical properties of oxide copper heap soils. Minerals Engineering. 2018, 117: 100–107.

[15] Dhawan N, Safarzadeh M S, Miller J D, et al. Crushed ore agglomeration and its control for heap leach operations. Minerals Engineering. 2013, 41: 53–70.

[16] Bouffard S C. Agglomeration for heap leaching: Equipment design, agglomerate quality control, and impact on the heap leach process. Minerals Engineering. 2008, 21(15):1115–1125.

[17] Thenepalli T, Chilakala R, Habte L, et al. A brief note on the heap leaching technologies for the recovery of valuable metals. Sustainability. 2019, 11(12): 3347.

[18] Wang L, Yin S, Deng B. Understanding the Effect of Stepwise Irrigation on Liquid Holdup and Hysteresis Behavior of Unsaturated Ore Heap. Minerals. 2021, 11(11): 1180.

[19] Zhang Y, Zhang B, Yang S, et al. Enhancing the leaching effect of an ion-absorbed rare earth ore by ameliorating the seepage effect with sodium dodecyl sulfate surfactant. International Journal of Mining Science and Technology. 2021, 31(6): 995–1002.

[20] Ai C, Sun P, Wu A, et al. Accelerating leaching of copper ore with surfactant and the analysis of reaction kinetics[J]. International Journal of Minerals, Metallurgy, and Materials. 2019, 26(3): 274–281.

[21] Jin J, Lin C L, Miller J D, et al. X-ray Computed Tomography Evaluation of Crushed Copper Sulfide Ore for Pre-concentration by Ore Sorting. Mining, Metallurgy & Exploration. 2022, 39(1): 13–21.

[22] Ai C M, Sun P P, Wu A X, et al. Liquid spreading behavior and surface wettability of copper ore under controlled sulfuric acid solution and surfactants. Journal of Central South University. 2022,29(2): 433–442.

[23] Wu A X, Ai C M, Wang Y M, et al. Influence of surfactant on permeability of heap leaching of copper ore [J]. Journal of Central South University (Science and Technology). 2014, 45(3): 895–901.

[24] Wang X, Wang H, Sui C, et al. Permeability and Adsorption–Desorption Behavior of Rare Earth in Laboratory Leaching Tests. Minerals. 2020, 10(10): 889.

[25] Zhou L, Wang X, Huang C, et al. Development of pore structure characteristics of a weathered crust elution-deposited rare earth ore during leaching with different valence cations. Hydrometallurgy. 2021, 201: 105579.

[26] Rinck-Pfeiffer S, Ragusa S, Sztajnbok P, et al. Interrelationships between biological, chemical, and physical processes as an analog to clogging in aquifer storage and recovery (ASR) wells. Water Research. 2000, 34(7):2110–2118.

